# Anodal Transcranial Direct Current Stimulation Reduces Competitive Anxiety and Modulates Heart Rate Variability in an eSports Player

**DOI:** 10.2174/17450179-v18-e2209270

**Published:** 2022-09-30

**Authors:** Sergio Machado, Leandro de Oliveira Sant’Ana, Bruno Travassos, Diogo Monteiro

**Affiliations:** 1 Department of Sports Methods and Techniques, Federal University of Santa Maria, Santa Maria, Brazil; 2 Department of Sports Science, University of Beira Interior, Covilhã, 6201-001 , Portugal; 3 Laboratory of Physical Activity Neuroscience, Neurodiversity Institute, Queimados, 26325-020, Brazil; 4 Postgraduate Program in Physical Education, Federal University of Juiz de Fora, Juiz de Fora, Brazil; 5 Research Center in Sport, Health and Human Development (CIDESD), Vila Real 5000-558, Portugal; 6 Portugal Football School, Portuguese Football Federation, Cruz 1495-433, Quebrada, Portugal; 7 Life Quality Research Center (CIEQV), Rio Maior, 2040-413, Portugal; 8 ESECS, Polytechnic of Leiria, Leiria, 2411-901 , Portugal

**Keywords:** Anxiety, Dosrsolateral prefrontal cortex, Heart rate variability, HRV, Transcranial direct current stimulation, tDCS

## Abstract

Previous research has recently shown that high cognitive and somatic anxiety and low self-confidence, before and during sport competitions have a significant correlation with heart rate variability (HRV) changes and can reduce overall athletic performance. Therefore, interventions, such as transcranial direct current stimulation (tDCS), can be a potential tool to reduce psychophysiological anxiety-related and enhance athletic performance. We present a case of a male professional athlete of eSports. We explored the effects of a single session of anodal tDCS (a-tDCS) at 2mA over the dosrsolateral prefrontal cortex (DLPFC) on competitive anxiety and HRV assessed in baseline (BL), pre-tDCS, post-tDCS and post-game moments and compared between moments. Here, we found a decrease in somatic and cognitive anxiety, as well as an increase in self-confidence and in SDNN index in the post-tDCS moment compared with BL, pre-tDCS and post-game moments. These findings can be a result of an acute change in the attentional state, influencing the processing of threatening information essential for cognitive anxiety and of a self-regulatory process, which can regulate physiological arousal response, such as HRV.

## Dear Editor,

Electronic sports (eSports) depend much more on cognitive and psychological skills for sports performance than traditional sports [1]. Literature shows that competitive anxiety, as well as self-confidence, are extremely important factors in the context of eSports performance [2], however there is no study examining competitive anxiety as a determining factor in the final score of a game, as well as about the effects of transcranial direct current stimulation (tDCS) on competitive anxiety in eSports athletes [3]. In addition, previous research has recently shown that high cognitive and somatic anxiety and low self-confidence, before and during sports competitions have a significant correlation with heart rate variability (HRV) changes, which might provide useful insights into the individual psychophysiological responses associated with athletic performance [4]. To the best of our knowledge, there are no studies on the impact of tDCS on competitive anxiety and HRV in the eSports field. Therefore, interventions, such as tDCS, can be a potential tool to modulate anxiety-related psychophysiological responses and enhance athletic performance [3, 5].

tDCS is a non-invasive brain stimulation technique which delivers weak electric currents (1–2 mA) using two electrodes applied to the scalp to induce prolonged changes in cortical excitability even after the end of the stimulation [6, 7]. The anodal current increases the cortical excitability, favoring the depolarization of the neuronal membrane, whereas the cathodal current has an inhibitory effect, causing hyperpolarization of the neuronal membrane [8]. Depending on the intensity and duration of the electric current imposed through the tDCS, these effects can last for more than an hour [8]. tDCS does not produce action potentials in the target brain areas, on the contrary, tDCS modulates the resting potential of the neuronal membrane, leading to changes in synaptic transmission [9].

In general, professional athletes deal with several stressors during a competition, which can affect emotional and anxiety states [10]. Competitive anxiety and self-confidence are especially important in the context of sports and can be a determining factor in the final outcome of a competition [11]. Before a competition [12], for example, several brain areas are activated and inhibited by structures of the limbic system, such as the medial prefrontal cortex, the hippocampus and the amygdala, which send multisynaptic afferents to the brainstem and hypothalamic activators of the brain to hypothalamic-pituitary-adrenocortical (HPA) axis and the autonomic nervous system (ANS) [13]. Due to tDCS being considered a neuroenhancement tool, since tDCS has been shown improvement in neurocognitive functions [3, 14] and changes in heart rate variability responses [15], we believe that a-tDCS applied to the dorsolateral prefrontal cortex (DLPFC) regulate anxiety and autonomic control, since DLPFC is one of the main areas responsible for emotional regulation and psychophysiological responses [16].

Therefore, a 20 years old male professional athlete with 6 years of experience in the practice of the game Counter Strike: Global Offensive was recruited, and no neuropsychiatric or osteoarticular diseases, nor use of any caffeine drink, smoke, alcohol or drugs on the day before the experiment was allowed. The Player was exposed to a decisive game in CS:GO PGL Major Fall championship. The experiment respected the Helsinki declaration and occurred just after the player’s signed the informed consent form. Thus, 48 hours before the game, the player was familiarized with the Revised Competitive State Anxiety Inventory - 2 (CSAI-2R) and the HRV measurements. Twenty-four hours before the game (*i.e*., baseline moment - BL) the player responded to the CSAI-2R [17], and HRV has recorded at rest for 10 minutes [18]. Thirty minutes before the tDCS application (*i.e*., pre-tDCS moment) and immediately after tDCS application (*i.e*., post-tDCS moment) before the beginning of the game, CSAI-2R and 10 minutes of HRV recording was performed by the same researcher. Both sessions were carried out in the game house between 14:00-17:00h to avoid circadian effects on psychological and autonomic performance and were conducted by the same researcher. The player sat in a comfortable chair for HRV and CSAI-2R acquisition and to receive a-tDCS.

Considering that a-tDCS over the left DLPFC administered at 2 mA for 20-30 min with electrodes between 9 and 25 cm^2^ improves acutely the core neurocognitive functions (*i.e*., working memory, decision making, attention, and multitasking) [3], thus, a-tDCS was administered at 2 mA for 20 min using a pair of pads embedded in saline (NaCl 140 mmol dissolved in milli-q water) comprising the two electrodes of 25 cm^2^ placed on the scalp using elastic bands, connected to a stimulator (TCT, Hong Kong, China). The anodal electrode was placed vertically over the left DLPFC located in the F3 electrode area, and the cathode was also placed vertically over the right orbitofrontal cortex (OFC) located in the FP2 electrode according to the international system of 10-20 EEG [19].

**Fig. (1):**
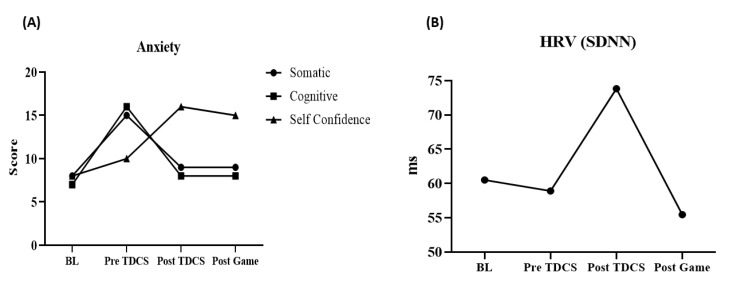
Anxiety and heart rate variability behavior before and after a competitive game of eSports. Note: (**A**) Behavior of subtypes of anxiety throughout study; (**B**) Behavior of heart rate variability throughout study; BL: baseline; Pre-tDCS: moment before tDCS application; Post-tDCS: moment after tDCS application; Post-game: moment after the end of the game.

Lower values can be observed in the post-tDCS compared to the values in the BL, pre-tDCS and post-game considering CSAI-2R, for the somatic and cognitive components. For the self-confidence component, higher values can be observed in post-tDCS compared to values in BL, pre-tDCS and post-game (Fig. **1A**). HRV data was recorded at rest for 10 minutes during the pre-and post-game (RS800 Precision Performance version 4.01.029, Polar, Finland). HRV was imported into a specific software (KUBIOS HRV - HEART RATE VARIBILITY ANALYSIS - VERSION 3.0.2, 2017), and analyzed under the moment domain, extracting the following derivative data only for SDNN (standard deviation of normal-to-normal RR ranges) [9]. SDNN reflects all the cyclic components responsible for the variability in the heart rate during recording [18], thus, it influences both sympathetic and parasympathetic components of the autonomic nervous system. The increase in SDNN values means that parasympathetic activity is predominant over sympathetic activity, howev er when the opposite occurs (*i.e*., reduced values of SDNN) there is a predominance of sympathetic activity over parasympathetic activity [19]. SDNN was higher at post-tDCS compared to values in the BL, pre-tDCS and post-game (Fig. **1B**).

Regarding the tolerability to the tDCS application, the player reported some mild effects through the adverse effects questionnaire [20] (*i.e*., scalp pain, tingling, itching, burning sensation, and redness of the skin) during the tDCS session. This questionnaire explains if a participant had experienced any adverse event and his relationship with the tDCS session. The questionnaire is composed of categorical issues with a score ranging from 0 to 5, being 0 - none, 1 - very mildly, 2 - mildly, 3 - moderate, 4 - severe, 5 - very serious, and was applied after the tDCS session to check the occurrence of 13 symptoms during or after the tDCS session.

Regarding psychological responses to competition, and according to our expectations, our findings are corroborated by Mehrsafar *et al*. [21] that revealed an acute decrease in somatic and cognitive anxiety after a-tDCS was applied over the DLPFC, but unlike us, self-confidence remained unaffected. Literature shows that athletes with greater self-confidence are the ones that best manage their stress in competitive conditions [22]. Thus, the application of a-tDCS over the left DLPFC appears to have positively influenced on self-confidence at the post-tDCS moment before the beginning of the competitive game. These findings can be a result of a self-regulatory process, which can regulate physiological arousal responses, such as HRV [23]. In addition, previous studies have shown that a-tDCS applied over the DLPFC acutely altered the attentional state, which influences the processing of threatening information essential for cognitive anxiety [24]. Evidence indicated that the top-down regulation of negative emotions, such as anxiety, was associated with increased activity in the left DLPFC and decreased in the right DLPFC [25], which justifies our findings through a-tDCS applied over the left DLPFC.

With regard to mechanisms underlying tDCS, the literature is still scarce, and more evidence is necessary. We speculate that the application of the a-tDCS over DLPFC led to an increase in parasympathetic modulation (*i.e*., vagal system) or to a reduction of sympathetic modulation (*i.e*., activation of amygdala and insula) [25]. Due to ventromedial pre-frontal cortex (VMPFC) and OFC are located below DLPFC and their involvement in affective/emotional processing is likely that a possible adjacent modulation may have influenced anxiety [26]. Therefore, tDCS may have contributed to an autonomic balance observed by the increase in SDNN, which means a predominance of parasympathetic activity over sympathetic activity [26], and to an emotional regulation demonstrated by the improvement in overall anxiety, which probably contributed to a good performance in the game.

As limitations, case reports are exploratory and cannot allow generalizable findings due to the lack of randomization, control group and sham stimulus. However they open new perspectives to create new hypotheses, and lead to subsequent investigations with other designs. Therefore, we suggest that a-tDCS could be used in a sample with a larger number of eSport players. Moreover, the use of non-focal tDCS may have influenced other cortical areas, which makes it difficult to ascertain whether the present results were exclusively due to the isolated stimulation of the DLPFC.
